# Public stigma against family members of people with mental illness: findings from the Gilgel Gibe Field Research Center (GGFRC), Southwest Ethiopia

**DOI:** 10.1186/1472-698X-14-2

**Published:** 2014-02-21

**Authors:** Eshetu Girma, Anne Maria Möller-Leimkühler, Norbert Müller, Sandra Dehning, Guenter Froeschl, Markos Tesfaye

**Affiliations:** 1 Department of Health Education and Behavioral Sciences, Jimma University, Jimma, Ethiopia; 2 CIHLMU Center for International Health, Ludwig-Maximilians-Universität, Munich, Germany; 3 Department of Psychiatry and Psychotherapy, Ludwig-Maximilians-Universität, Munich, Germany; 4 Department of Infectious Diseases and Tropical Medicine, Ludwig-Maximilians-Universität, Munich, Germany; 5 Department of Psychiatry, Jimma University, Jimma, Ethiopia

**Keywords:** Stigma, Mental illness, Family stigma, Psublic stigma

## Abstract

**Background:**

Public stigma against family members of people with mental illness is a negative attitude by the public which blame family members for the mental illness of their relatives. Family stigma can result in self social restrictions, delay in treatment seeking and poor quality of life. This study aimed at investigating the degree and correlates of family stigma.

**Methods:**

A quantitative cross-sectional house to house survey was conducted among 845 randomly selected urban and rural community members in the Gilgel Gibe Field Research Center, Southwest Ethiopia. An interviewer administered and pre-tested questionnaire adapted from other studies was used to measure the degree of family stigma and to determine its correlates. Data entry was done by using EPI-DATA and the analysis was performed using STATA software. Unadjusted and adjusted linear regression analysis was done to identify the correlates of family stigma.

**Results:**

Among the total 845 respondents, 81.18% were female. On a range of 1 to 5 score, the mean family stigma score was 2.16 (±0.49). In a multivariate analysis, rural residents had significantly higher stigma scores (std. β = 0.43, P < 0.001) than urban residents. As the number of perceived signs (std. β = -0.07, P < 0.05), perceived supernatural (std. β = -0.12, P < 0.01) and psychosocial and biological (std. β = -0.11, P < 0.01) explanations of mental illness increased, the stigma scores decreased significantly. High supernatural explanation of mental illness was significantly correlated with lower stigma among individuals with lower level of exposure to people with mental illness (PWMI). On the other hand, high exposure to PWMI was significantly associated with lower stigma among respondents who had high education. Stigma scores increased with increasing income among respondents who had lower educational status.

**Conclusions:**

Our findings revealed moderate level of family stigma. Place of residence, perceived signs and explanations of mental illness were independent correlates of public stigma against family members of people with mental illness. Therefore, mental health communication programs to inform explanations and signs of mental illness need to be implemented.

## Background

In the work of Goffman, the stigma against family members of people with mental illness (PWMI) is described as “courtesy or associative stigma, which is the process by which a person is stigmatized by virtue of association with another stigmatized individual” [1]. Larson et al. described it as; “family stigma contains the stereotypes of blame, shame, and contamination; public attitudes which blame family members for incompetence may conjure the onset or relapse of a family member’s mental illness” [2]. Although stigmatization of family members’ may not be necessarily due to the stigmatizing of the patients, studies have found that family members reported feelings of stigma, i.e. the report of family members’ experience of stigma, could be attributed to either actual or perceived stigma from the public [2-7].

A frequently observed reason for stigma against family members of PWMI was related to the explanations for mental illnesses [2]. As evidenced by previous studies, whether people have biogenetic, psychosocial (‘poor’ parenting/care) and/or supernatural explanations of mental illness can be associated with stigma against PWMI [8,9]. The other common reason for public stigma against family members of people with mental illness was the incrimination that families failed to help their relatives with mental illness to adhere to a recommended treatment [2,10].

Both supernatural and non-supernatural explanations of mental illness may lead to family stigma. As a result, the public may develop less contact to the patients. Less contact of the public with the patients and not disclosing about the mental illness situation of the patient were found to be associated with stigmatization of the patients [11-13]. The latter may also finally lead to stigmatization of family members.

Quantitative and qualitative findings suggested that when the public holds negative attitude towards the family members of PWMI, the family may resort to social self restrictions. The family may also hide their sick relative, which in turns may lead to delay in treatment seeking, and discrimination from getting services. All of these may result in poor quality of life, depression and increased emotional burden on families [2,3,14-18].

To combat such consequences and challenges, there are effective interventions such as educating the public, contact to the patients (not hiding the patients from the community and integrating them to the community system) and empowering the patients and families in order to reduce stigma associated with patients and family members [19-26].

Although the key role of family members in care provision in mental health is well appreciated and an accepted concept, family stigma is under researched and this study is the first of its kind in Ethiopia. Therefore, this study has attempted to generate baseline data on the situation of stigma for further studies and interventions in the Gilgel Gibe Field Research Center (GGFRC), Southwest Ethiopia. The study aimed at investigating the extent and correlates of public stigma against family members of PWMI in the study area. It was hypothesized that the study population mean stigma would be more than the mean stigma (2.5) score and the psychographic (such as perceived explanations, signs, etc.) and socio-demographic (example: age, sex, residency, etc.) were expected correlates of family stigma.

## Methods

The cross-sectional house to house survey was conducted among randomly selected 845 urban and rural community members in the GGFRC, Southwest Ethiopia. The GGFRC is Demographic Surveillance Site (DSS) and has been recording and storing data on vital events and socioeconomic parameters since its establishment in May 2005. Studies ranging from molecular level to population surveys have been conducted in GGFRC by Jimma University in collaboration with other partners. In 2011, 54, 538 persons were living in the center [27]. It is a field research center for the Health Sciences Research Institute of Jimma University. The study participants were selected using a simple random sampling technique from the household list in the Health Sciences Research Institute of Jimma University. The data was collected through face-to-face interviews using structured questionnaires by trained interviewers. Trained and experienced personnel who were working in the GGFRC supervised the data collection. The details of the sampling procedures can be obtained freely from a previous publication of the same project about stigma against people with mental illness [28]. The previous study can be also accessed freely by anyone using the PubMed Central Identification (PMCID) of PMC3853185.

Family stigma was measured using 10 items with Likert scale (1 = strongly disagree to 5 = strongly agree) responses adapted from Devaluation of Consumer Families Scale and other two previous studies [10,29,30]. The tool included items related to avoiding social interaction with family members of people with mental illness, blaming the family members for the mental illness of their relatives, undermining the family members of people with mental illness, the need for controlling their family member who is mentally ill behind closed doors and not to disclose about their family member’s mental illness to others. Example of the items include: “I believe that parents of children with a mental illness are not as responsible and caring as other parents”. Reversely oriented items were reverse coded before data analysis. The overall family stigma was computed by summing-up the scores on all of the ten items. Accordingly, a higher score indicated a higher public stigma against family members of PWMI.

In addition to the scale of stigma against family members of PWMI, measures on socio-demographic and psychographic characteristics were included in the questionnaire. The psychographic characteristics included (a) 3 items measuring perceived supernatural (example: evil spirit), (b) 6 items measuring non-supernatural (biological and psychosocial) explanations of mental illness (example: stress and drug addiction), (c) 8 items measuring exposure to people with mental illness (PWMI) (example: message from TV/radio, ever worked or lived with people with mental illness) and (d) 12 items measuring perceived signs (example: suicide attempt, self neglect and sleep disturbance) of mental illness, and were measured as yes = 1 and no = 0 scores. After summing up scores on the respective psychographic characteristic, higher values indicated higher perceived supernatural, psychosocial and biological explanations, perceived signs, and exposure to PWMI. The questionnaire was translated into Amharic and Afaan Oromo languages and then back translated into English. Translation and back-translation was done to ensure semantic equivalence. After pre-testing, the final questionnaire was administered either in Amharic or Afaan Oromo languages based on the respondents language ability.

Before data entry, each questionnaire was checked for completeness and consistency. Data entry was done by using EPI-DATA version 3.1. The data was then exported to STATA version 10.0 for analysis. Normality of the stigma against family members of people with mental illness score was checked using histograms and kernel density. Since the stigma score was not normally distributed, logarithmic transformation was done. After the transformation, the distribution of stigma score was normal. Then, for categorical independent variables, the mean stigma scores were compared using ANOVA and *t* tests. For continuous independent variables, correlation tests were done to check for their association with stigma score. Finally, unadjusted and adjusted linear regression models were developed to identify the correlates of stigma against family members of PWMI. Standardized regression coefficients were presented for variables which were found significant in the bivariate analysis. A p-value less than 0.05 was used to declare statistical significance in the bivariate and multivariate analysis. Tolerance analysis (variance inflation factor) was done for checking multicollinearity between variables. Subsequently, interaction analysis was performed to explore the effects of the interactions between variables with multicollinearity.

Ethical approval was obtained from Research Ethics Review Board of Jimma University. Written permission was granted by Health Sciences Research Institute, Jimma University. Finally, written informed consent was obtained from the individual participants before the interviews.

## Results

### Socio-demographic characteristics

A response rate of 100% was achieved in this study. Among the total 845 respondents, 517 (81.18%) were female and 638 (75.50%) of them ever been married. The mean age (standard deviation) was 37.4 (±14.8) years. The majority of respondents were Muslims (88.99%) and members of Oromo ethnic group (91.12%). Nearly two-thirds of the respondents (62.72%) were illiterate. Most of the respondents (80.00%) were farmers. The households’ average monthly income (standard deviation) was 377.3 (±392.5) ETHB (1USD ≈ 18.5ETHB) and the average family size (standard deviation) was 5.2 (±2.2) (Table 1).

**Table 1:** Socio-demographic characteristics of respondents in GGFRC, south west Ethiopia, 2012 (N = 845)

**Variable**	**Frequency**	**Percent**
**Sex**		
Female	517	61.18
Male	328	38.82
**Marital status**		
Ever been married^*^	638	75.50
Never been married	207	24.50
**Religion**		
Muslim	752	88.99
Others (Orthodox, Protestant)	93	11.01
**Ethnicity**		
Oromo	770	91.12
Others^***^	75	8.88
**Educational status**		
Could not read and write	530	62.72
Read and write only	96	11.36
Elementary and above	219	25.92
**Occupation**		
Farmer and house wife	676	80.00
Others^**^	169	20.00

^*^Single, divorced and widowed, ^**^private work, Student, government employee, House worker (maid), ^***^Yem, Guraghe, Amhara, Keffa and Dawro.

### Belief and perception about mental illness

Six hundred thirty-six (75.27%) believed that mental illnesses can be cured. A very small proportion (1.66%) of the respondents ever had a history of mental illness, and 9.70% ever had a relative with a history of mental illness. On a range of 0–8 scores, the mean exposure to PWMI was 1.9 (±1.2). The mean number of reported signs of mental illness was 2.8 (±1.2) on a 0–12 range. The average number of perceived supernatural explanations of mental illness score was 0.6 (±0.7) on a 0–3 range while the average number of perceived psychosocial and biological explanations of mental illness score was 1.7 (±0.9) on a 0–6 range.

### Stigma against family members of people with mental illness scores

As depicted in Table 2, among the ten items measuring family stigma, the highest mean stigma score (2.81 ± 1.23) was found for the item which stated that ‘families who have a member with mental illness ought to be treated differently than other families’. The second highest mean stigma score (2.43 ± 1.07) was found for the item which stated ‘parents of children with mental illness are not just as responsible and caring as other parents’. The third highest mean score (2.24 ± 1.05) was on the item ‘people should keep their family member with mental illness behind locked doors’.

**Table 2:** Mean score of items measuring family stigma in GGFRC, south west Ethiopia, 2012

**Item**	Possible scores^*^	**Mean**	**SD**
Families with a member who is mentally ill should be treated in the same way they treat other families (reverse coded)	1-5	2.81	1.23
I believe that parents of children with a mental illness are not just as responsible and caring as other parents	1-5	2.43	1.07
People should keep their family member with mental illness behind locked doors	1-5	2.24	1.05
Families with a member of serious mental illness should not be visited as often as families without mental illness	1-5	2.21	0.98
Parents of children with mental illness should be blamed for the mental illness of their children	1-5	2.18	1.13
It would be foolish to marry a family member of a man/woman who has suffered from mental illness	1-5	2.13	1.05
I do not feel good to be friends with families that have a relative who is mentally ill living with them	1-5	2.09	1.00
Families with a member of serious mental illness should be ashamed of them selves	1-5	1.99	1.04
People should never tell to anyone that they have a family member with mental illness	1-5	1.94	0.85
Families with a member of mental illness should not be allowed to be a member of social gatherings and institutions	1-5	1.63	0.85
Overall score	1-5	2.16	0.49

^*^(1 = strongly disagree, 2 = disagree, 3 = neutral, 4 = agree, 5 = strongly agree).

The overall mean family stigma score was 2.16 (±0.49) on a range of 1 to 5 score (Table 2). Statistically significant differences in family stigma score were observed between rural and urban, between religions, among ethnic groups and different types of occupation. Family stigma was found to have significant negative correlations with educational level, family income, perceived signs, and perceived psychosocial and biological explanation of mental illness (P < 0.05). On the other hand, significant positive correlation was observed between family stigma and perceived supernatural explanation of mental illness (P < 0.05) (Table 3).

**Table 3:** Mean score of family stigma based on socio-demographic backgrounds in GGFRC, south west Ethiopia, 2012

**Variables**	**Mean**	**SD**	***t*-test (ANOVA)**	**P-value**
**Sex**				
Female	2.16	0.49	0.00	0.95
Male	2.17	0.49		
**Living with partner**				
Ever been married	2.18	0.49	1.47	0.23
Never been married	2.13	0.51		
**Setting**				
Rural	2.30	0.50	177.63	<0.001
Urban	1.87	0.29		
**Religion**				
Muslim	2.19	0.49	15.19	<0.001
Others	1.98	0.44		
**Ethnicity**				
Oromo	2.19	0.49	27.93	<0.001
Others	1.88	0.38		
**Educational status**				
Could not read and write	2.24	0.50	25.20	<0.001
Read and write only	2.22	0.51		
Elementary and above	1.97	0.42		
**Occupation**				
Farmer and house wife	2.22	0.49	44.00	<0.001
Others	1.95	0.41		

### Predictors of public stigma against family members of people with mental illness

All the variables that showed statistically significant association in the bivariate analyses (*t* test, ANOVA or correlation) were entered into a multivariate linear regression analysis for controlling possible confounders. Based on the analysis, residency (rural, urban), the number of perceived signs of mental illness, perceived supernatural, as well as perceived psychosocial and biological explanations of mental illness were found to be independent predictors of family stigma. Except residency, other socio-demographic characteristics were not significantly correlated with stigma in a multivariate analysis.

Rural residents exhibited significantly higher stigma scores (std. β = 0.43, P < 0.001) than urban residents. Residency was also the strongest predictor of public stigma against family members of PWMI. As the number of reported perceived signs of mental illness increased, family stigma decreased significantly (std. β = -0.07, P < 0.05). Both higher perceived supernatural (std. β = -0.12, P < 0.01), and psychosocial and biological (std. β = -0.11, P < 0.01) explanations of mental illness were significantly associated with lower family stigma (Table 4). Over all, the model explained 21.07% of the variance of public stigma against family members of PWMI. The scale used to measure family stigma had a reliability coefficient (Cronbach’s alpha) of 0.70.

**Table 4:** Predictors of family stigma in GGFRC, south west Ethiopia, 2012

**Variables**	**Unadjusted β (standardized)**	**Adjusted β (standardized)**
**Rural**	0.42^***^	0.43^***^
**Muslim**	0.14^***^	-0.05
**Oromo**	0.19^***^	0.07
**Educational level**	-0.23^***^	-0.03
**Farmer and housewife**	0.23^***^	-0.01
**Family average income**	-0.10^**^	0.04
**Perceived signs of mental illness**	-0.15^***^	-0.07^*^
**Perceived supernatural explanations**	0.08^*^	-0.12^**^
**Perceived psychosocial and biological explanations**	-0.19^***^	-0.11^**^

^*^P < 0.05, ^**^P < 0.01, ^***^P < 0.001.

### Interaction effects

**Figure 1:**
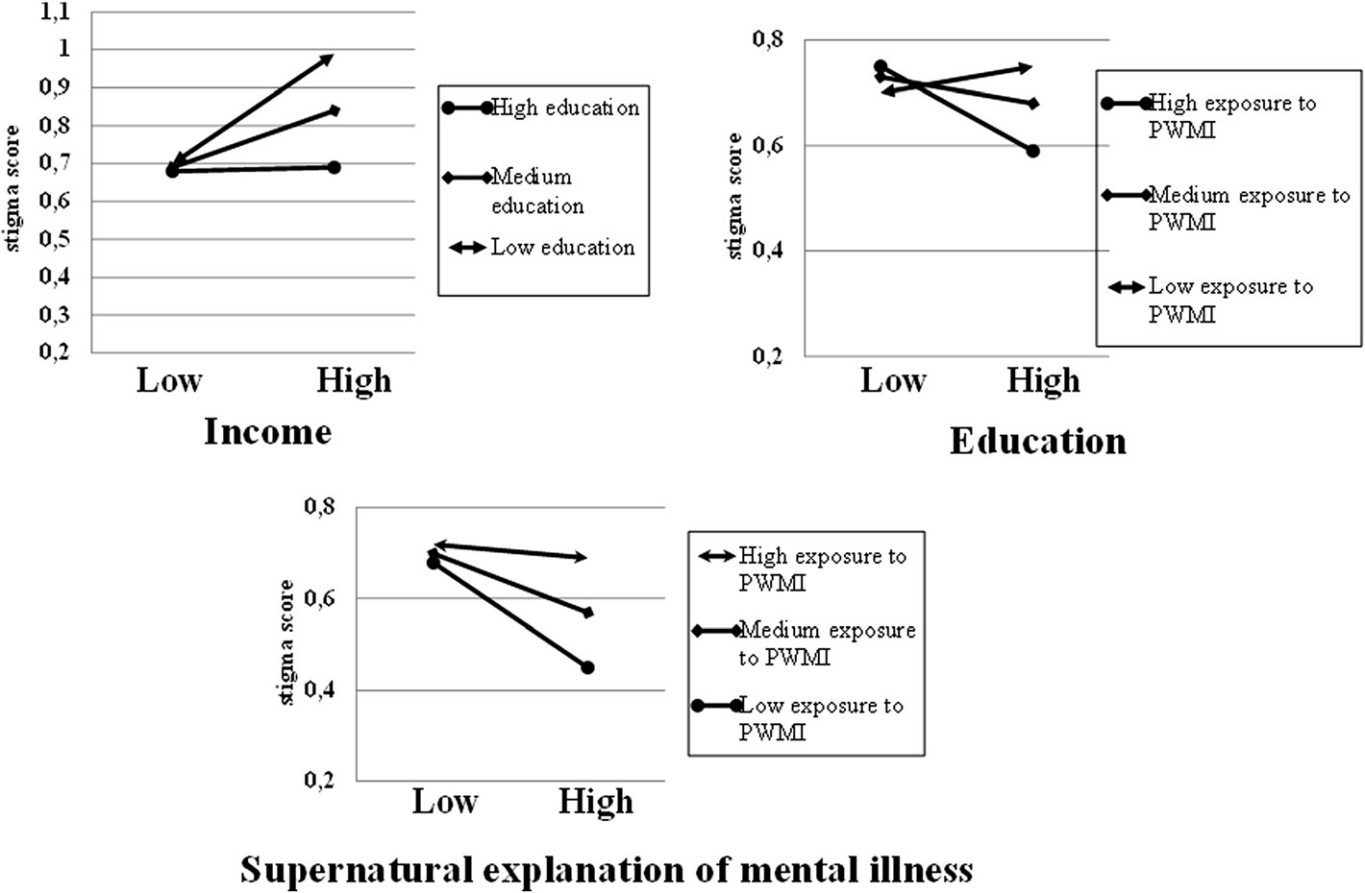
Family stigma score at different levels of education and exposure to mental illness with respect to income, education and perceived supernatural explanation of mental illness scores in the Gilgel Gibe Field Research Center, Southwest Ethiopia, 2012.

After checking the presence of multicollinearity among predictor variables, interaction analysis was performed. Accordingly, significant interaction was found between education and income, education and exposure to PWMI, and exposure to PWMI and perceived supernatural explanations of mental illness. Then, a separate analysis was done after controlling the effects of other variables. As the income of a respondent increased, the perceived family stigma increased significantly at both medium (std. β = 0.15, P < 0.01) and low education (std. β = 0.29, P < 0.001) levels. As education increased, significant lower family stigma (std. β = -0.16, P < 0.01) was found at high exposure to PWMI. At both medium (std. β = -0.13, P < 0.01) and lower (std. β = -0.23, P < 0.001) levels of exposure to PWMI, significant lower public stigma was scored as the supernatural explanation of mental illness increased (Figure 1).

## Discussion

We found the overall family stigma in the community to be of moderate level. Furthermore, living in rural place, explanations regarding the cause of mental illness, perceived signs of mental illness were associated with family stigma. However, living in rural place was the strongest predictor of high family stigma.

The moderate level of family stigma in the current study can be directly or indirectly associated with the public stigma against PWMI or due to low mental illness information as found in the current study. A previous study in the same study area reported that there was high public sigma against PWMI [28]. Nonetheless, the current score was lower compared to the stigma against PWMI score reported in the previous study [28].

Rural residents have shown significantly high stigma than urban residents which may be due to low mental health literacy and rural respondents may be disadvantaged of other underlying causes such as high illiteracy, low media and mental health service access which implies that reducing the gap on such determinants may enhance reducing of stigma against family members of PWMI.

One of the reasons of stigma development is lack of explanation and fear about a given illness [1,31]. Similarly, in the current study both high perceived supernatural and psychosocial and biological explanations of mental illness were significantly correlated with lower stigma against family members of PWMI. This indicates that there is high need for programs targeted at increasing the public awareness about the causes and nature of mental illness to reduce stigma against family members of PWMI.

High supernatural explanation of mental illness was associated with lower stigma at lower level of exposure to PWMI. This can be related to the type of explanation and sympathy that people with high supernatural but lower exposure to PWMI might have i.e. they may be less likely to blame the family for the relatives’ mental illness. Similarly, significantly lower stigma was obtained when individuals scored high on exposure to people with mental illness at high education level. This may be due to the combination of high education level which can facilitate exposure to diverse media on mental illness and enhance the ability to understand messages related to PWMI.

High number of reported signs of mental illness by the public was significantly correlated with lower stigma against family members of PWMI. Similarly, stigma against PWMI was lower among people who were familiar to the illness, and those who had previous contact to persons with mental illness [19-21,25,32-34]. People who are aware of many signs of mental illness may have better general information about mental illness through formal and informal means. Thus, they may have also less stereotyped beliefs and prejudices.

Respondents who had high income but low education showed significantly high family stigma. Such type of respondents may be in a disadvantage to get more information about mental illness from other sources like print and visual media. In addition, they may also have limited opportunity to get awareness and knowledge about mental illness from the school environment.

Generally, in the current study there was a high tendency of blaming family members for the illness of the patients. The belief among the public for the need to restrict the patients by the family members to avoid contact to the community may be associated with the type of explanation of mental illness and perceived dangerousness of people with mental illness. On the other hand, a low score was observed on restricting family members from being a member of social gatherings. In the multivariate analysis, no significant correlation was scored between many socio-demographic characteristics (i.e., age, sex, marital status, religion, ethnicity and occupation) and stigma against PWMI. Exposure to PWMI was very low in the current study which calls for mental health awareness interventions in the study community.

This study is the first of its kind exploring family stigma in Ethiopia. The relatively large randomly selected community sample representing diverse social and economic background adds to the robustness of our data. Although we have achieved semantic equivalence of the measurement, the lack of other aspects of validation could be potential limitation. In addition, the face-to-face interviews, which were most appropriate in the context of high level of illiteracy, may have resulted in social desirability bias while responding stigma items. Nevertheless, our findings contribute to the existing body of knowledge regarding the correlates of family stigma in low-income setting.

## Conclusion

There is a moderate level of family stigma in the southwest Ethiopia (GGFRC). Explanations of mental illness held by the public whether supernatural or non-supernatural predict lower level of public stigma against family members of PWMI. Supernatural explanations can reduce stigma significantly at lower level of exposure to PWMI and persons with mental illness. Previous exposure to PWMI reduces stigma significantly among people with high level of education. Similarly, being familiar to the signs and symptoms of mental illness also may reduce public stigma against family members of PWMI. Since public stigma may affect the family members and the patients negatively, mental health communication programs aimed at raising awareness about the causes and signs of mental illness need to be implemented with special focus on rural communities. Increasing contact to PWMI as well as their family members also may be helpful in reducing public stigma against family members of PWMI.

## Competing interests

The authors declare that they have no competing interests.

## Authors’ contributions

EG, SD and MT designed the study, involved in the data collection, analysis and drafting of the manuscript. NM, GF, AML were involved in the design of the study, analysis of the data and critically reviewed the manuscript. All authors read and approved the final manuscript.

## Pre-publication history

The pre-publication history for this paper can be accessed here:

http://www.biomedcentral.com/1472-698X/14/2/prepub
